# The Establishment of an Assay to Measure DNA Polymerase-Catalyzed Repair of UVB-Induced DNA Damage in Skin Cells and Screening of DNA Polymerase Enhancers from Medicinal Plants

**DOI:** 10.3390/ijms17050667

**Published:** 2016-05-04

**Authors:** Sawako Ikeoka, Tatsuo Nakahara, Hiroyasu Iwahashi, Yoshiyuki Mizushina

**Affiliations:** 1 Research Center, Maruzen Pharmaceuticals Co., Ltd., Onomichi, Hiroshima 722-0062, Japan; s-ikeoka@maruzenpcy.co.jp (S.I.); t-nakahara@maruzenpcy.co.jp (T.N.); h-iwahashi@maruzenpcy.co.jp (H.I.); 2 Graduate School of Agriculture, Shinshu University, Minamiminowa-mura, Kamiina-gun, Nagano 399-4598, Japan

**Keywords:** DNA polymerases, enzyme enhancer, normal human epidermal keratinocytes (NHEK), ultraviolet B (UVB), rose myrtle, piceatannol

## Abstract

An *in vitro* assay method was established to measure the activity of cellular DNA polymerases (Pols) in cultured normal human epidermal keratinocytes (NHEKs) by modifying Pol inhibitor activity. Ultraviolet (UV) irradiation enhanced the activity of Pols, especially DNA repair-related Pols, in the cell extracts of NHEKs. The optimal ultraviolet B (UVB) exposure dose and culture time to upregulate Pols activity was 100 mJ/cm^2^ and 4-h incubation, respectively. We screened eight extracts of medicinal plants for enhancement of UVB-exposed cellular Pols activity using NHEKs, and found that rose myrtle was the strongest Pols enhancer. A Pols’ enhancement compound was purified from an 80% ethanol extract of rose myrtle, and piceatannol was isolated by spectroscopic analysis. Induction of Pol activity involved synergy between UVB irradiation and rose myrtle extract and/or piceatannol. Both the extract and piceatannol reduced UVB-induced cyclobutane pyrimidine dimer production, and prevented UVB-induced cytotoxicity. These results indicate that rose myrtle extract and piceatannol, its component, are potential photo-protective candidates for UV-induced skin damage.

## 1. Introduction

Skin, the largest human organ has numerous functions including protection against physical, chemical, and biological attacks including ultraviolet (UV) radiation and microorganisms. Skin diseases are caused by a number of environmental and genetic factors, particularly chronic exposure of skin to UV radiation from the sun, which induces various responses including skin related effects (sunburn, hyperpigmentation, keratosis, elastosis, skin cancer), and immune effects (immunosuppression and acute inflammatory responses) [1,2]. Ultraviolet B (UVB) (290–320 nm) radiation induces keratinocyte apoptosis, which presents as sunburn in epidermal cells, the formation of which can be used to determine the severity of DNA damage. The absorption of UV by the skin causes two forms of DNA damage, cyclobutane pyrimidine dimmers (CPDs), and pyrimidine photoproducts. However, the accumulation of damaged cells in UVB-exposed skin is prevented by the presence of DNA damage repair mechanisms [3].

DNA-dependent DNA polymerase (Pol) (E.C. 2.7.7.7) is involved in DNA synthesis, replication, repair, and genetic recombination. Fifteen mammalian Pols have been identified to date, and their functions are currently being investigated [4,5]. Eukaryotic Pols are characterized into four families based on their sequence homology: A, B, X, and Y [5]. Family A includes mitochondrial Pol γ, θ and ν; family B includes DNA replicative Pols α, δ, ε, and ζ; family X contains Pols β, λ, μ, and terminal-deoxynucleotidyl transferase (TdT); and family Y consists of Pols η, ι, κ, and REV1 [5]. Pols α, δ, and ε are involved in DNA replication, Pol β has a role in base excision repair, Pol γ functions in mitochondrial replication and repair, Pols λ, μ, and TdT mediate non-homologous end-joining and immunological diversity, and Pols η, κ, ζ, REV1 mediate tolerance to DNA damage as well as translesion synthesis. Of note, certain Pols can be involved in multiple DNA processing pathways. Pol ζ and REV1 have a major role in translesion DNA synthesis (TLS) [5]. Of note, another TLS Pol, Pol η, is resistant to UV radiation-induced DNA damage, allowing the highly efficient repair of TT-*cis*-syn CPDs (TT-CPDs).

Harmful UV radiation effects on skin can be prevented or reduced by the use of sunscreens [6], although these might not be sufficient to prevent skin photodamage [7]. Recently, non-sunscreen compounds have been used to prevent UV-mediated skin damage [8]. The main features of these compounds should be the promotion of skin defense mechanisms or inhibition of pathological pathways. Previous studies reported that plant extracts such as polyphenols might protect skin against UV-induced damage [9,10].

The aim of this study was to identify components from medicinal plants that might enhance UV-damaged DNA repair-related Pols. Using natural product screens, we previously identified >100 low molecular weight organic compounds, including Pol inhibitors [11,12]. We also generated an *in vitro* assay for screening of mammalian Pol inhibitors [13,14]. Based on our previous studies, an *in vitro* Pol enhancer assay was established using cell extracts from cultured normal human epidermal keratinocytes (NHEKs). Eight medicinal plant extracts were screened for Pol enhancer activity on cultured NHEKs. Activity-enhancing compounds were purified from extracts of the most bioactive plants. Here, we report a relationship between cellular Pol activity and DNA repair in NHEKs with UVB-stimulated DNA damage when treated with bioactive plant extract compounds.

## 2. Results

### 2.1. Cellular Polymerase (Pol) Activity in Ultraviolet B (UVB)-Irradiated Normal Human Epidermal Keratinocytes (NHEKs)

**Figure 1:**
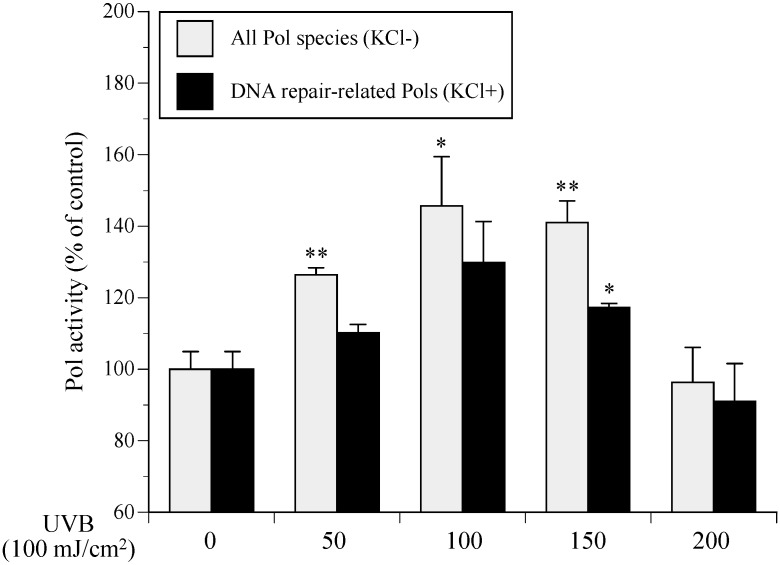
Cellular polymerase (Pol) activity in ultraviolet B (UVB)-exposed normal human epidermal keratinocytes (NHEKs) is dependent on UVB irradiation levels (50–200 mJ/cm^2^). After UVB irradiation, NHEKs were cultured for 4 h. Gray bars represent human whole Pol (standard reaction conditions without 120 mM KCl) and black bars represent DNA repair-related Pol species (with 120 mM KCl), respectively. Pol activity of vehicle control without UVB irradiation was arbitrarily designated 100%. All data are the mean ± SEM (*n* = 3). * *p* < 0.05 and ** *p* < 0.01 compared with the UVB (−) vehicle control.

When NHEKs were incubated for 4 h after UVB exposure, 50–150 mJ/cm^2^ of UVB resulted in upregulated Pol activity (Figure 1). Among the 15 human Pols, salt (KCl and NaCl) inhibited the activity of DNA replicative Pols (Pols α, δ, and ε) [4,5], whereas the activity of DNA repair-related Pol species (Pol families X and Y) were enhanced by salt (120 mM KCl). Activities of the purified calf Pol α and rat Pol β in the presence of 120 mM KCl were one-tenth lower and 1.5-fold higher, respectively, than in the absence of KCl (data not shown). Therefore, the standard reaction mixture for “all Pol species” containing both DNA replication and repair Pols (gray bars in Figure 1), or “DNA repair-related Pols” only, was tested in the presence of absence of 120 mM KCl (black bars in Figure 1). In the standard reaction mixture without salt (all Pol species) cellular Pol activity was higher than for the standard reaction mixture with salt (DNA repair-related Pols). Therefore, DNA replicative Pols (Pols α, δ and ε) and UV-damaged DNA repair Pols (Pols β and η) were enhanced in NHEKs following UVB exposure. Irradiation at 100 mJ/cm^2^ induced the greatest increase in Pol activity by 1.30- and 1.46-fold for the standard reaction mixtures with or without KCl, respectively. Interestingly, most cells had died when NHEK Pol activity was decreased at 200 mJ/cm^2^ (data not shown).

**Figure 2:**
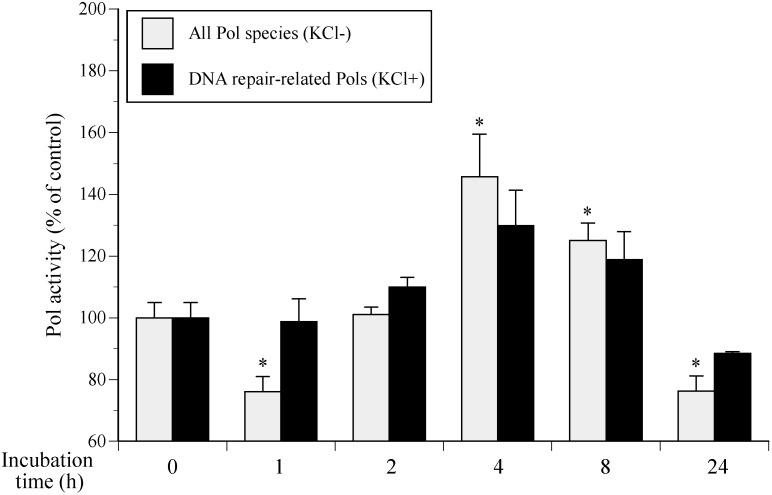
Pol activity depends on incubation time (1–24 h) in 100 mJ/cm^2^ UVB-exposed NHEKs. Gray bars represent human whole Pol (standard reaction conditions without 120 mM KCl) and black bars represent DNA repair-related Pol species (with 120 mM KCl), respectively. Pol activity of vehicle control without UVB irradiation was arbitrarily designated 100%. All data are the mean ± SEM (*n* = 3). * *p* < 0.05 with the UVB (−) vehicle control.

We investigated the effect of incubation time on Pol activity following 100 mJ/cm^2^ of UVB irradiation. Pol activity was highest in NHEKs incubated for 4 h among cells cultured over 1–24 h, indicating UVB-damaged DNA repair activity peaked 4 h after irradiation (Figure 2). Therefore, the most suitable NHEK culture conditions to increase Pol activity are 100 mJ/cm^2^ of UVB exposure and incubation for 4 h.

### 2.2. Medicinal Plant Pol Activity Enhancer Screen in UVB-Irradiated NHEKs

**Figure 3:**
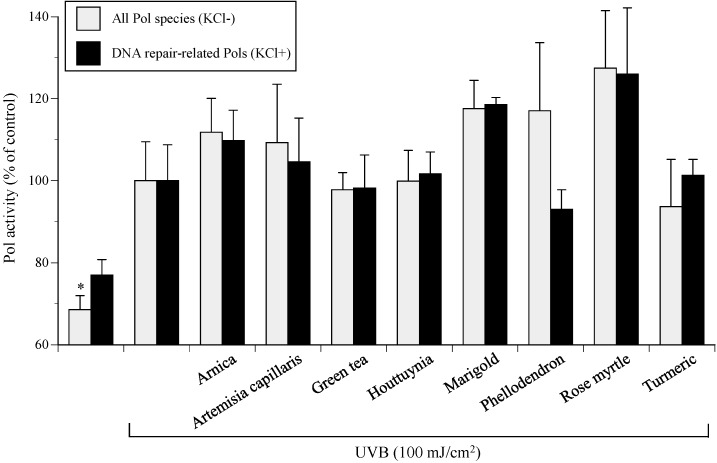
Effects of the extracts of eight medicinal plants on the activity of cellular Pols in UVB-irradiated NHEKs. NHEKs were cultured with each extract (10 μg/mL) for 24 h, NHEKs were then cultured for 4 h after UVB (100 mJ/cm^2^) irradiation, and the cellular Pol activity in the treated NHEKs was then measured. Gray bars represent human whole Pol (standard reaction conditions without 120 mM KCl) and black bars represent DNA repair-related Pol species (with 120 mM KCl), respectively. Vehicle control Pol activity with UVB irradiation was arbitrarily designated 100%. All data are the mean ± SEM (*n* = 3). * *p* < 0.05 compared with UVB^+^ vehicle control.

UVB-induced Pol active compounds were screened from eight extracts of medicinal plants (arnica, artemisia capillaris, green tea, houttuynia, marigold, phellodendron, rose myrtle, and turmeric). Activities of all Pol species and DNA repair-related Pols in cultured NHEKs were moderately enhanced by UVB irradiation and were further increased in the presence of 10 µg/mL of medicinal plant extracts (Figure 3). Rose myrtle extract was the strongest Pol activity stimulator in UVB-exposed NHEKs and showed 163% and 186% increased activity for standard reaction mixtures with or without KCl, respectively. Although the standard error of the mean (SEMs) of marigold extract were much tighter than those of rose myrtle extract, the Pol activity was higher for rose myrtle extract, which was used for all further experiments.

### 2.3. Effect of Rose Myrtle Extract on Cellular Pol Activity in UVB-Irradiated NHEKs

**Figure 4:**
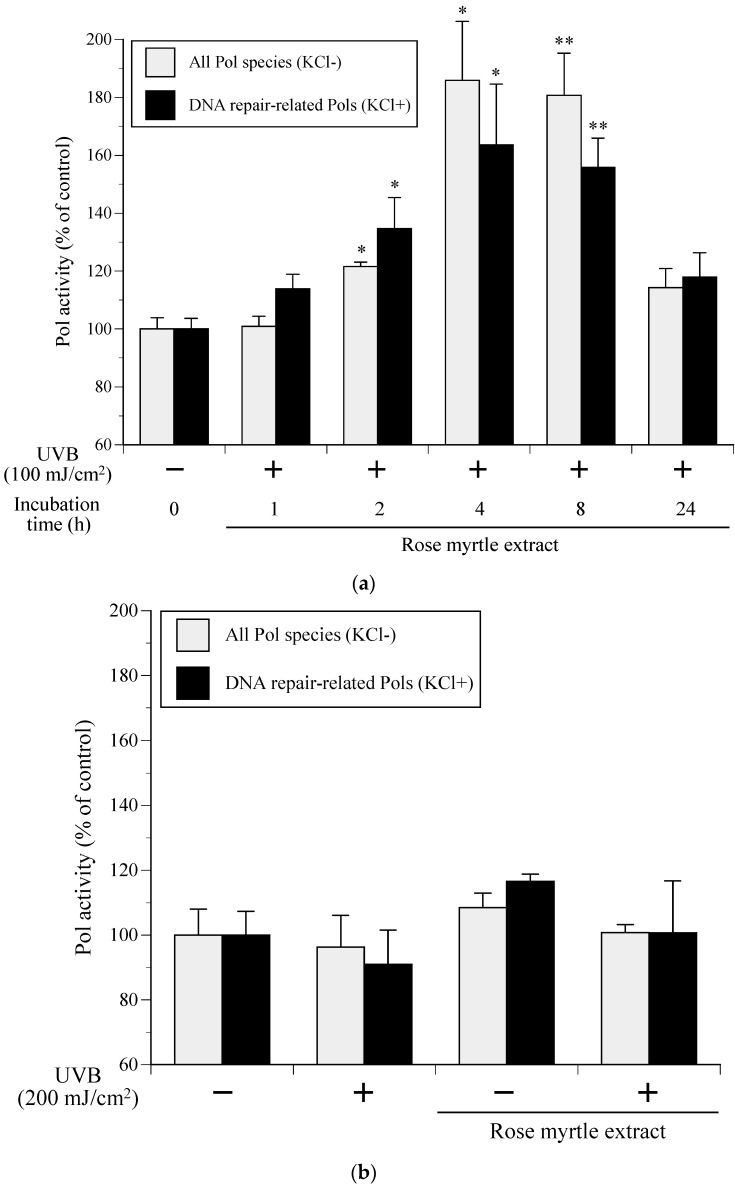
Effects of rose myrtle extract and UVB irradiation on the activity of cellular Pols in NHEKs. (**a**) NHEKs were cultured with rose myrtle extract (10 μg/mL) for 24 h, and then Pol activity was dependent upon incubation time (1–24 h) irradiated with 100 mJ/cm^2^ UVB; (**b**) NHEKs were cultured with or without rose myrtle extract (10 μg/mL) for 24 h, irradiated with or without an excess amount of UVB (200 mJ/cm^2^), and then incubated for 4 h. Cellular Pol activity in treated NHEKs was measured. Gray bars represent human whole Pol (standard reaction conditions without 120 mM KCl) and black bars represent DNA repair-related Pol species (with 120 mM KCl), respectively. Vehicle control Pol activity without UVB irradiation was arbitrarily designated 100%. All data are the mean ± SEM (*n* = 3). * *p* < 0.05 and ** *p* < 0.01 compared with UVB^−^ vehicle control.

After NHEKs were cultured with rose myrtle extract (10 μg/mL) for 24 h and then exposed to UVB, the cellular Pol activity in NHEKs, which were incubated for various times (1–24 h), was measured. As shown in Figure 4A, the Pol activity in the rose myrtle extract-treated and 100-mJ/cm^2^-UVB-irradiated NHEKs was the highest after 4 h of incubation, with 1.64- and 1.86-fold increases for standard reaction mixtures with or without KCl, respectively.

Next, we investigated whether the cellular Pol activity in NHEKs was affected by an excess amount of UVB (200 mJ/cm^2^) radiation and rose myrtle extract (10 μg/mL) treatment. When NHEKs were exposed to 200 mJ/cm^2^ of UVB (in the absence of rose myrtle extract treatment), the activities of all Pol species and DNA repair-related Pols decreased by 3.7% and 9.0%, respectively (Figure 4B). In contrast, cellular Pol activity moderately increased by culture with rose myrtle extract (without UVB irradiation) for 4 h incubation, and was not affected by both UVB radiation and rose myrtle extract treatment. These results suggested that the extract did not assist in the repair of the excessive DNA damage that occurred from the excessive UVB exposure.

### 2.4. Isolation of a Cellular Pol Component from Rose Myrtle Extract Active against UVB-Irradiated NHEKs

**Figure 5:**
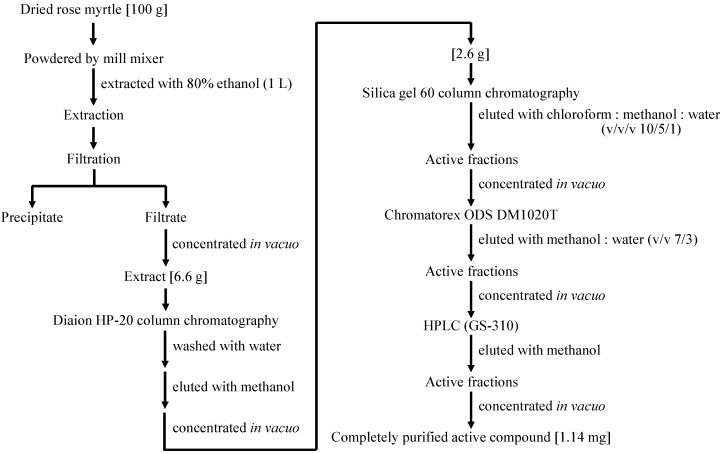
Purification procedure of Pol enhancer form rose myrtle.

For further tests, a Pol stimulated compound was purified from rose myrtle as follows. One hundred grams of rose myrtle was extracted with 80% ethanol (1 L). Then, 6.6 g of evaporated extract dissolved in distilled water underwent hydrophobic column chromatography (Diaion HP-20). The column was washed with water, and the methanol fraction was collected and then evaporated (2.6 g). This fraction underwent silica gel 60 column chromatography and was eluted with chloroform:methanol:water (*v*:*v*:*v*, 10:5:1). The obtained active fraction was purified using high-performance liquid chromatography (HPLC) with a reverse-phase silica gel column, eluted with methanol. A white powder (1.14 mg) was obtained (Figure 5).

**Figure 6:**
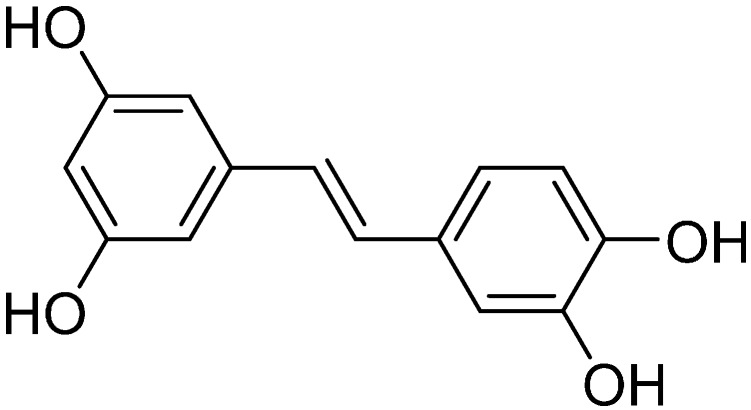
Structure of piceatannol.

The completely purified active compound was identified as piceatannol, a polyphenol (Figure 6), based on high-resolution mass spectrometry and a comparison of its ^1^H and ^13^C nuclear magnetic resonance data with previously published spectroscopic data [15]. The 80% ethanol extract of rose myrtle and purified piceatannol (purity of 98% determined by NMR analysis, data not shown) was used for further experiments.

### 2.5. Effect of Rose Myrtle Extract and Piceatannol on Pol Activity in UVB-Irradiated NHEKs

**Figure 7:**
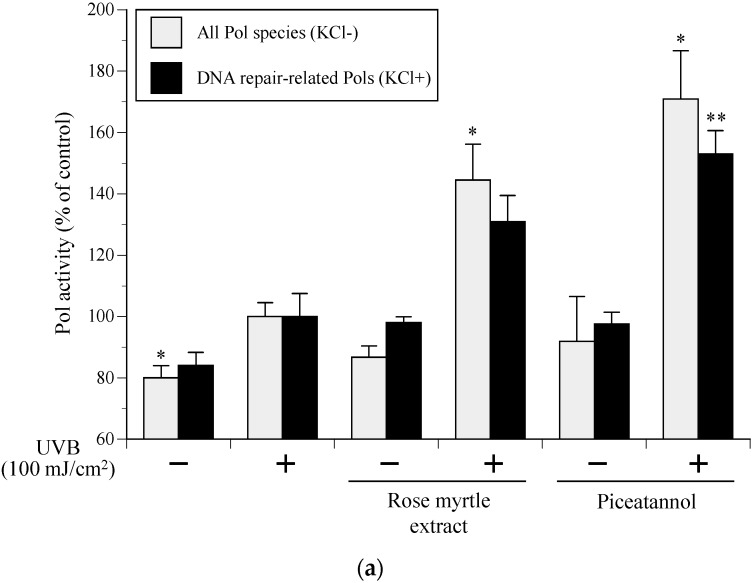

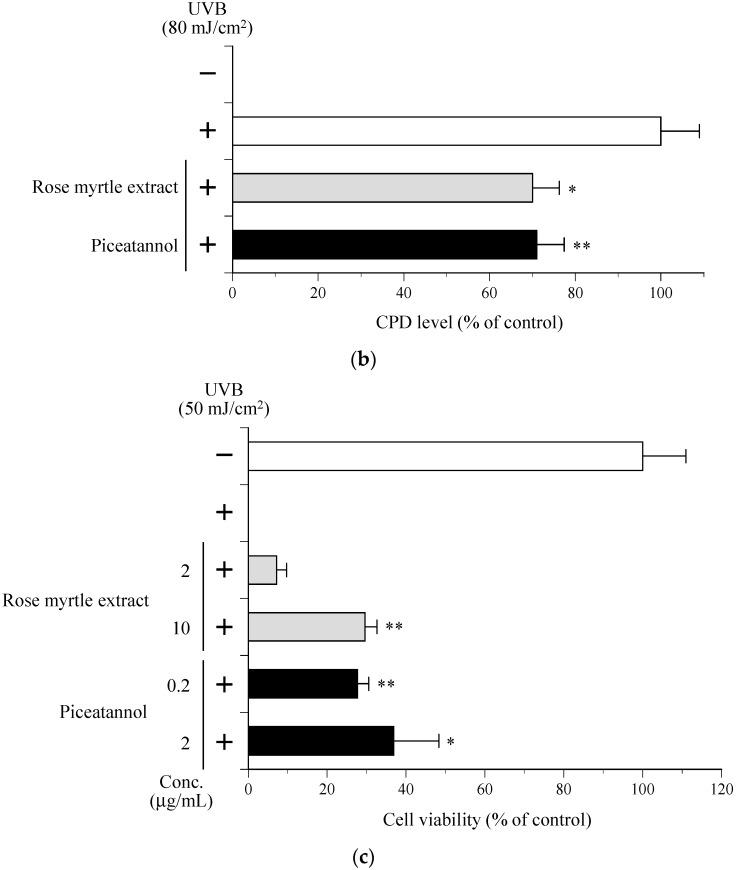
Effect of piceatannol purified from rose myrtle extract as a Pol enhancer on the bioactivities of UVB-exposed NHEKs. (**a**) The activity of cellular Pols. NHEKs were cultured with or without rose myrtle extract (10 μg/mL) or piceatannol (2.0 μg/mL) for 24 h, and then NHEKs were cultured for 8 h after UVB (100 mJ/cm^2^) irradiation. Cellular Pol activity in the treated NHEKs was measured. Gray bars represent human whole Pol (standard reaction conditions without 120 mM KCl), and black bars represent DNA repair-related Pol species (with 120 mM KCl), respectively. Pol activity of vehicle control without UVB irradiation was arbitrarily designated 100%. All data are expressed as mean ± SEM (*n* = 3). * *p* < 0.05 and ** *p* < 0.01 compared with the UVB^+^ vehicle control; (**b**) UVB-induced CPD production. Before UVB (80 mJ/cm^2^) irradiation, NHEKs were incubated with rose myrtle extract (10 μg/mL) or piceatannol (2.0 μg/mL). Quantitative evaluation of CPDs by DNA-ELISA was performed. Vehicle control CPD production with or without UVB irradiation was 100% or 0%, respectively. All data are expressed as mean ± SEM (*n* = 6). * *p* < 0.05 and ** *p* < 0.01 compared with UVB^+^ vehicle control; (**c**) Cell viability of NHEKs irradiated with UVB (50 mJ/cm^2^), and treated with rose myrtle extract (2 and 10 μg/mL) or piceatannol (0.2 and 2 μg/mL). MTT [3-(4,5-dimethylthiazol-2-yl)-2,5-diphenyl tetrazolium bromide] assays evaluated living cell numbers at 24 h after treatment. Vehicle control cell viability with or without UVB irradiation was arbitrarily designated 0% or 100%, respectively. All data are the mean ± SEM (*n* = 6). * *p* < 0.05 and ** *p* < 0.01 compared with the UVB^+^ vehicle control.

In the absence of compound treatment, UVB exposure at 100 mJ/cm^2^ increased the activities of all Pol species and DNA repair-related Pols by approximately 125% and 119%, respectively. In the UVB-exposed NHEKs, 10 µg/mL of rose myrtle extract and 2.0 µg/mL of piceatannol significantly enhanced NHEK Pol activity by 1.56–1.81-fold and 1.82–2.14, respectively (Figure 7A). There was synergy between UVB irradiation and rose myrtle extract and/or piceatannol on the induction of Pol enzyme activity because rose myrtle extract had little effect on the cellular Pol activity in non-UVB-exposed NHEKs (Figure 4B).

### 2.6. Effect of Rose Myrtle Extract and Piceatannol on Cyclobutane Pyrimidine Dimmer (CPD) Production in UVB-Irradiated NHEKs

CPD formation is characteristic of DNA damage and mutagenesis [16]. We investigated whether rose myrtle extract or piceatannol influenced the removal of CPDs from DNA in UVB-irradiated NHEKs. Exposure of NHEKs to 80 mJ/cm^2^ UVB-induced CPD formation immediately after irradiation, and this was used as a reference for DNA damage (Figure 7B). CPD levels were measured after UVB exposure to determine DNA repair in irradiated cultures. Controls consisted of the non-repaired reference sample. Both rose myrtle extract (10 µg/mL) and piceatannol (2.0 µg/mL) decreased CPD production by approximately 30% in UVB-exposed NHEKs compared with non-treated control cells. Therefore, DNA repair activity of UVB-damaged DNA in NHEKs might be mediated by rose myrtle extract and/or piceatannol.

### 2.7. Effect of Rose Myrtle Extract and Piceatannol on Cell Viability in UVB-Irradiated NHEKs

Cell viability of NHEKs was measured 24 h after UVB irradiation (50 mJ/cm^2^) and compared with non-treated cells. Cell viability was 80% greater in the 50 µg/mL rose myrtle extract cells and 30% greater in piceatannol (0.2 µg/mL)-treated cells compared with non-treated cells (Figure 7C). Of note, rose myrtle extract contained 0.2% piceatannol, indicating that piceatannol is an active component of rose myrtle extract that protects against UVB-induced cell death.

## 3. Discussion

Here, we report the establishment of a Pol activator *in vitro* assay using purified cell extracts from UVB-exposed cultured NHEKs (Figure 1 and Figure 2) to identify enhanced cellular Pol in 80% ethanol extracts from rose myrtle and piceatannol (Figure 3, Figure 4, Figure 5, Figure 6 and Figure 7) [17]. Although Pols synthesize DNA and are critical for genome duplication, they also protect cells against DNA damage from numerous sources including water-catalyzed reactions, reactive oxygen species that inflict continual damage, and ubiquitous causes of lesions such as solar ionizing radiation. UV radiation introduces DNA intra-strand cross-linked CPDs, a four-member ring structure generated from pyrimidine 5,6 double-bond saturation, which distorts DNA to inhibit replicative Pols and stall DNA replication fork progression [18]. Several DNA repair mechanisms, such as DNA nucleotide excision repair (NER), that remove damaged DNA to reduce the risk of replicative Pols encountering DNA lesions have been identified. DNA NER mechanisms recognize and repair bulky DNA adducts such as CPDs [19], and genetic defects in NER are associated with a rare autosomal recessive disease called xeroderma pigmentosum (XP) [20], which is characterized by an early onset of freckling, sun sensitivity, photophobia, and neoplastic changes of sun-exposed skin. Of note, Pol η purified from human HeLa cells restored the replication of DNA containing CPD lesions from the cell extracts of XP variant cells [21]. Adenine deoxynucleotides is correctly inserted at opposite linked bases of a TT-CPD by purified human Pol η [22]. Enhanced UV-induced genetic instability was induced by Pol β, a base excision repair (BER) Pol, which enabled the translesion replication of CPDs in a UV lesion bypass [23]. Therefore, activation of DNA repair-related Pols β and η might reduce UVB-induced DNA damage.

Solar UV radiation, especially UVB, might cause approximately 90% of skin inflammation cases as it is efficiently absorbed by cellular DNA [24]. Direct and indirect DNA damage is caused by UVB radiation that penetrates the skin epidermis. Here, we showed that rose myrtle extract and/or piceatannol removed CPD photoproducts (Figure 7B) and increased UVB-exposed NHEK cell viability (Figure 7C), indicating enhanced DNA damage repair. The most predominant skin DNA lesions caused by UVB and UVA exposure are CPDs and 6-4 pyrimidine-pyrimidine photoproducts [16,25]. NER is the main UVB-induced DNA damage repair mechanism. Following skin cell exposure to excessive UV radiation, CPD lesions remain because the NER capacity is reduced, ultimately causing cell death, mutagenesis, senescence, and/or skin carcinogenesis [25]. In conclusion, the current study indicated rose myrtle extract and piceatannol are protective against UVB-irradiated NHEK death and sun-damage in UVB-irradiated human skin explants by enhancing pre-existing DNA repair mechanisms.

## 4. Materials and Methods

### 4.1. Materials

Eight extracts of medicinal plants, arnica (trade name: ARNICA EXTRACT BG), artemisia capillaris (trade name: ARTEMISIA CAPILLARIS EXTRACT BG), green tea (trade name: GREEN TEA EXTRACT), houttuynia (trade name: HOUTTUYNIA EXTRACT), marigold (trade name: MARIGOLD EXTRACT), phellodendron (trade name: PHELLODENDRON EXTRACT BG-J), rose myrtle (trade name: ROSE MYRTLE EXTRACT BG80), and turmeric (trade name: TURMERIC EXTRACT BG) were obtained from Maruzen Pharmaceuticals Co., Ltd. (Hiroshima, Japan). The solid contents of these extracts were used for experiments. The following materials were purchased from KURABO Industries Ltd. (Osaka, Japan): NHEK and serum-free keratinocyte growth medium (KGM, trade names: EpiLife-KG2 and HuMedia-KG2) containing growth additives (bovine pituitary extract, human epidermal growth factor) and hydrocortisone, insulin, and gentamycin/amphotericin B. A chemically synthesized DNA template, poly(dA), was from Sigma-Aldrich Inc. (St Louis, MO, USA), and customized oligo(dT)_18_ DNA primers were from Sigma-Aldrich Japan K.K. (Hokkaido, Japan). The radioactive nucleotide [^3^H]-labeled 2′-deoxythymidine-5′-triphosphate (dTTP; 43 Ci/mmol) was obtained from Moravek Biochemicals Inc. (Brea, CA, USA). All other reagents were analytical grade and were purchased from Nacalai Tesque Inc. (Kyoto, Japan).

### 4.2. Cell Culture

NHEKs, seeded at a density of 6 × 10^5^ cells/cm^2^ into 75-cm^2^ cell culture flasks, were cultured in KGM at 37 °C in 5% CO_2_ in air. The passage number of the NHEKs was 1. Compounds to be tested were dissolved in dimethyl sulfoxide (DMSO) and then diluted with medium to an appropriate concentration. The final volume was adjusted to 0.05% (*v*/*v*) DMSO.

### 4.3. Measurement of Cellular Pol Activity in NHEKs

NHEKs were grown to sub-confluence in 60-mm^2^ culture dishes (7.5 × 10^5^ cells/5 mL) with KGM. They were then treated for 24 h with test compounds. Cultures were washed with Hank’s buffer, UVB-irradiated (0–200 mJ/cm^2^), and cultured in KGM for 0–24 h. Cultured cells were then collected by cell scraping, sonicated in lysis buffer containing 50 mM Tris–HCl (pH 7.5), 1 mM EDTA, 5 mM 2-mercaptoethanol, 15% glycerol, and a protease inhibitor cocktail of Complete Mini (Roche Diagnostics, Mannheim, Germany) for 10 s using a sonicator (model, UR-20P; TOMY SEIKO Co., Ltd., Tokyo, Japan; sonication level, low). Cell extract Pol activity was assayed and quantified *in vitro* as described [13,14] with modifications.

Poly(dA)/oligo(dT)_18_ and [^3^H]-dTTP were used as DNA template-primer substrate and nucleotide (dNTP; 2′-deoxynucleotide-5′-triphosphate) substrate, respectively for Pol reactions. Standard reaction mixtures for all Pol species contained 50 mM Tris–HCl, pH 7.5, 10 µM [^3^H]-dTTP (100 cpm/pmol), 1 mM MgCl_2_, 1 mM dithiothreitol, 5 µM poly(dA)/oligo(dT)_18_ (A/T, 4:1), and 15% (*v*/*v*) glycerol. The DNA repair-related Pol species standard reaction mixture was the same, but also contained 120 mM KCl. After 37 °C incubation for 60 min, radioactive DNA products were collected by a DEAE–cellulose paper disc (DE81) as described previously [26]. Radioactivity was measured with a scintillation counter (2300TR TriCarb; PerkinElmer, Downers Grove, IL, USA).

### 4.4. Purification and Identification of a Cellular Pol Enhancer

The cellular Pol stimulated compound was purified from the screened medicinal plant using various column chromatography, consisting of Diaion HP-20 (Sigma Aldrich, St. Louis, MO, USA) and silica gel 60 (Merck Millipore, Darmstadt, Germany), and HPLC, consisting of Chromatorex ODS DM1020T (Fuji Silysia Ltd., Durham, NC, USA). The purified compound was identified using high-resolution mass spectrometry (Xevo G2 Tof: Waters; Milford, MA, USA) and nuclear magnetic resonance equipment (JNM-ECS400: JEOL RESONANCE; Tokyo, Japan).

### 4.5. Measurement of CPD Production

Sub-confluent NHEKs in 60-mm^2^ culture dishes (2 × 10^5^ cells/2 mL) with KGM were treated for 24 h with test compound in KGM. Cultures were washed with Hank’s buffer, UVB-irradiated (80 mJ/cm^2^), and test compound was added in KGM for 6 h. After treatment, cultured cells were collected by cell scraping. Nuclear DNA was purified by QIAamp Blood Kit (Qiagen, Tokyo, Japan). CPD levels in the quantified DNA were measured by enzyme-linked immunosorbent assay (ELISA) with anti-CPD monoclonal antibody (1:1000; cat. no. NMDND001; Cosmobio Co., Ltd., Tokyo, Japan), according to the manufacturer’s instructions.

### 4.6. Measurement of Cell Viability

Sub-confluent NHEKs in KGM in 48-well plates (2 × 10^4^ cells/0.2 mL) were washed with Hank’s buffer, UVB-irradiated (50 mJ/cm^2^), and test compound treated in KGM for 24 h. Then, cell viability (*i.e.*, percent of living cells) was evaluated by MTT [3-(4,5-dimethylthiazol-2-yl)-2,5-diphenyl tetrazolium bromide] assay [27]. Absorbance of cells exposed to MTT at 2 h was measured at λ 570 nm with a μQuant plate reader (BioTek Instruments, Inc., Winooski, VT, USA). Simultaneously, absorbance at λ 650 nm was measured as turbidity. Differences between measurements were the amount of produced blue formazan.

### 4.7. Statistical Analysis

All data are expressed as the mean ± standard error of the mean (SEM) of at least three independent determinations per experiment. Statistical significance between each experimental group was analyzed by a Student’s *t*-test. Statistical significance was when *p* values of 0.001, 0.01, and 0.05 were obtained depending on experiment and comparison.

## 5. Conclusions

This study reports the development of a rapid and simple *in vitro* Pol activity screening technique containing UVB-irradiated NHEKs. Rose myrtle and piceatannol protected skin from UVB-induced damage by enhancing DNA repair-related Pol enzyme activity. Therefore, plant extracts containing Pol activity enhancing compounds may have potential as non-sunscreen derived cosmetics.

## Abbreviations

Pol	DNA polymerase
NHEKs	normal human epidermal keratinocytes
UV	ultraviolet
CPDs	cyclobutane pyrimidine dimmers
TT-CPDs	TT-*cis*-syn CPDs
TdT	terminal-deoxynucleotidyl transferase
TLS	translesion DNA synthesis
KGM	keratinocyte growth medium
dTTP	2′-deoxythymidine-5′-triphosphate
DMSO	dimethyl sulfoxide
dNTP	2′-deoxynucleotide-5′-triphosphate
ELISA	enzyme-linked immunosorbent assay
MTT	3-(4,5-dimethylthiazol-2-yl)-2,5-diphenyl tetrazolium bromide
SEM	standard error of the mean
NER	nucleotide excision repair
XP	xeroderma pigmentosum
BER	base excision repair
